# Doubly blind: a systematic review of gender in randomised controlled trials

**DOI:** 10.3402/gha.v9.29597

**Published:** 2016-04-15

**Authors:** Susan P Phillips, Katarina Hamberg

**Affiliations:** 1 Departments of Family Medicine and Public Health Sciences, Queen's University, Kingston, Canada; 2 Umeå Centre for Gender Studies, Umeå University, Umeå, Sweden; 3 Department of Public Health and Clinical Medicine, Family Medicine, Umeå University, Umeå, Sweden

**Keywords:** clinical trials, randomised controlled trial, gender, socio-economic status, social determinants, social epidemiology, sex differences

## Abstract

**Background:**

Although observational data show social characteristics such as gender or socio-economic status to be strong predictors of health, their impact is seldom investigated in randomised controlled studies (RCTs).

**Objective & design:**

Using a random sample of recent RCTs from high-impact journals, we examined how the most often recorded social characteristic, sex/gender, is considered in design, analysis, and interpretation. Of 712 RCTs published from September 2008 to 31 December 2013 in the *Annals of Internal Medicine*, *British Medical Journal*, *Lancet*, *Canadian Medical Association Journal*, or *New England Journal of Medicine*, we randomly selected 57 to analyse funding, methods, number of centres, documentation of social circumstances, inclusion/exclusion criteria, proportions of women/men, and reporting about sex/gender in analyses and discussion.

**Results:**

Participants’ sex was recorded in most studies (52/57). Thirty-nine percent included men and women approximately equally. Overrepresentation of men in 43% of studies without explicit exclusions for women suggested interference in selection processes. The minority of studies that did analyse sex/gender differences (22%) did not discuss or reflect upon these, or dismissed significant findings. Two studies reinforced traditional beliefs about women's roles, finding no impact of breastfeeding on infant health but nevertheless reporting possible benefits. Questionable methods such as changing protocols mid-study, having undefined exclusion criteria, allowing local researchers to remove participants from studies, and suggesting possible benefit where none was found were evident, particularly in industry-funded research.

**Conclusions:**

Social characteristics like sex/gender remain hidden from analyses and interpretation in RCTs, with loss of information and embedding of error all along the path from design to interpretation, and therefore, to uptake in clinical practice. Our results suggest that to broaden external validity, in particular, more refined trial designs and analyses that account for sex/gender and other social characteristics are needed.

## Introduction

Randomised controlled trials are thought to provide the strongest research evidence of clinical potential or efficacy for medical interventions. By randomly assigning subjects to intervention and control groups both the characteristics of interest but also those that are unidentified should be equally distributed across study arms, allowing researchers to eliminate the effect of individual and social characteristics not being studied.

To examine rather than control for the impact of social traits on health outcomes requires a very different approach. Socio-economic status (SES), race/ethnicity, sex/gender, or social connectedness, for example, must then be measured and considered as independent covariates that alter health outcomes. In reality social traits are not independent but act interdependently to shape opportunities and constraints that may alter gene expression, risk, compliance, access to care, and pathways from exposure to illness (1, 2). Study protocols and inclusion criteria should be designed accordingly with enrolment that is large enough to allow for disaggregated analyses of, for example, results for women and men. The strength of randomisation is that baseline although not necessarily static social determinants will be equally distributed and eliminated as sources of bias, while researchers manipulate or control exposures of interest. The weakness is that unless they are managed as variables for analysis the very real impact of those social circumstances on the study endpoint, and interactions with the intervention of interest are hidden. Resulting study findings will then speak only of efficacy in a population cleansed of personal traits and social circumstances, but not of effectiveness and external validity in the real world where no one is devoid of such characteristics as sex/gender or SES.

Lifetime fluctuations in and the social nature of circumstances like SES are readily apparent; however, placing sex and gender among these may require explanation. Sex and gender are two separate but intertwined terms used for categorisation and analyses of men and women. Sex refers to biological attributes and is primarily associated with physical and physiological features, including chromosomes, hormone function, and sexual anatomy. Gender goes beyond biology and refers to the socially constructed roles, behaviours, expressions, and identities of girls, women, boys, men, and gender diverse people. It influences how people perceive themselves and each other, how they act and interact, and the distribution of power and resources in society. Gender is not static and not something a person possesses, it is rather an activity. The concept of ‘doing gender’ manifests this. Doing gender most often incorporates, but can also challenge explicit and implicit social norms, constraints, and expectations that alter ways of behaving and acting as men and women (3–5). Like SES, gender will vary across settings and over time. Although the impact of a group or society's gender norms is not ubiquitous or homogeneous, there are commonalities arising from the experience of being, for example, a woman within a given grouping. Furthermore, like SES, gender can, although it does not always, affect health and wellbeing. For example, in many cultures girls are undervalued relative to boys and are therefore fed less. Similarly, women in many countries are less educated than men, not because of limited individual capacity but because societal and cultural norms imply that higher education is only for men. In countries where women are well educated, or even more educated than men, they are still often under-represented in high-ranked and well-paid jobs, suggesting that gender inequality is not restricted to developing countries but is a worldwide phenomenon. Women currently outlive men globally; however, their longevity advantage has and continues to fluctuate with time and social circumstances (6). This life expectancy difference likely arises from how men and women live their lives, which work and activities they engage in, and risks they are exposed to or take (7–9). Changes in other social circumstances may change the expectations of and roles consigned to men or to women and may accordingly change their doing of gender, illustrating that gender is not a fixed characteristic (5).

In reality, it is seldom possible to isolate sex from gender, as biology interacts with social and environmental living conditions, and sex and gender become tangled together. Genes can be activated or shut down (temporarily or permanently) by environmental factors and ways of living. Since men and women often live different lives with different duties, demands, and resources, this newer epigenetic knowledge contributes to insights about how gender and sex are intertwined. Therefore, using the terms sex and gender has to be done with caution. In this article, we use the term ‘sex-specific’ when talking about diseases or conditions that are restricted to either men or women, like prostate cancer or preterm birth. Gender is used as a separate term when we talk about bias related to being either women or men – because the creation of gender bias is by definition a social process whereby preconceptions and ideas about women and men skew research, investigations, or treatment. Sex or gender are also used as separate terms as the authors of a specific reviewed paper did so. However, we prefer sex/gender as a term that recognises that biology shapes social context which, in turn, shapes biology.

Within a randomly selected sample, exposures being examined may not have uniform or homogeneous effects across social groupings such as sex/gender, race or SES, of study participants (10). If variability is not individual but instead arises from a characteristic of a social subgroup, the statistical independence of participants will be jeopardised. Failure to recognise that subjects may not be independent will introduce error despite randomisation (10). Observational research has demonstrated strong and extensive health effects arising from and associated with membership in the groupings, ‘women’ and ‘men’. Significant sex/gender differences have been well documented with respect to, for example, heart diseases (11) and type 2 diabetes (12). Pharmacokinetics may differ for men and women, as can benefits, side effects, and adverse reactions to drugs (13). Unequal access to medical care for women and men in many settings is of importance in understanding treatment and health outcomes. Finally, gender bias has been demonstrated in clinical decision-making (14, 15). Although it does not always alter health, evidence is strong enough to justify considering sex/gender whenever possible, as a modifier of the relationship between intervention and medical outcome (16).

To directly study the impact of any characteristics on a particular outcome in experimental designs requires the ability to randomly allocate these to participants. Although not fixed, social characteristics cannot be randomly assigned. Nevertheless, it is possible to estimate how traits like sex/gender or SES alter outcomes rather than dismissing them as topics not worthy of study in a particular trial. At a minimum, examining interactions of these with independent variables will hint at their effect. Powering a study to enable a priori randomisation of recruited women and men separately so that the study intervention can be examined both within and across these groupings and to assess interactions of social determinants with other variables will increase accuracy and meaning (17–19).

The aim of this systematic sampling review of recent randomised controlled studies (RCTs) is to determine whether and how the social traits of sex/gender are addressed in design, analysis, and interpretation of study findings and to then consider the meanings and impact of the methodologies used. We selected sex/gender for specific examination because the categories ‘man’ and ‘woman’ were the most commonly identified social characteristics in the reviewed RCTs.

## Methods

### Sample selection

In September 2013, we searched PubMed using the terms randomised controlled trial, clinical trial, human, *Annals of Internal Medicine*, *British Medical Journal*, *Lancet*, *Canadian Medical Association Journal*, *New England Journal of Medicine* and the search filters clinical trial, September 2008–1 July 2013. We then sorted the initial 588 papers retrieved by date of publication to randomise papers from each journal and ensure sampling of the entire time frame, and selected every 20th paper for inclusion. In January 2014, a similar search with date limits of July 2013 to 31 December 2013 yielded another 124 papers, of which every fourth paper was selected for review. Recent papers were oversampled to ensure that findings reflected most current research methodology.

**Fig. 1:**
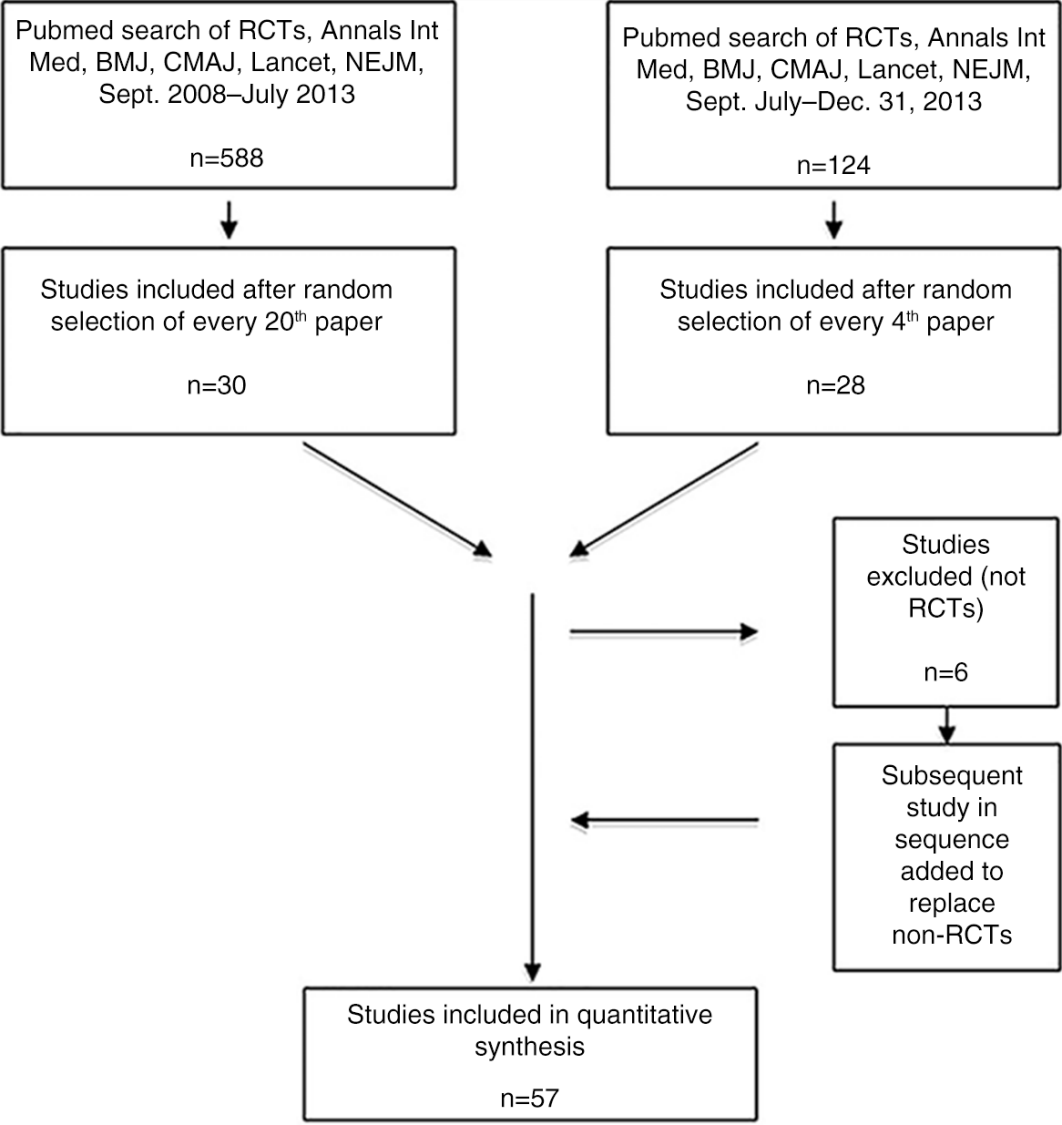
Flow diagram of study search, randomisation, and selection.

When a selected paper was not an RCT (*n*=6), the next paper on the list was substituted. To establish our analytical method and construct a data extraction template, five studies were reviewed by both researchers, then three more were reviewed independently, and thereafter discussed for concordance of data extraction. After each reviewing another 10 and 9 studies, respectively, both authors again checked for inter-reviewer consistency in approach, information extraction, and interpretation of findings, then reviewed 30 more papers (15 each) independently. All in all, 57 papers were included in the analysis. Sample selection is summarised in Fig. 1.

### Data extracted and analysis

Although many authors in the sample used the concepts ‘sex’ and ‘gender’ as interchangeable synonyms and without defining them when describing inclusion, exclusion, or effects of being men or women, we used the combined term sex/gender in our data extraction templates. Authors generally documented body mass index (BMI) only in studies of diseases where weight was an important factor in a biological sense. However, as BMI is also strongly related to and entangled with the level of education, family economy, and other aspects of SES, we included it as a social factor when extracting data. We noted inclusion/exclusion criteria for each social category (sex/gender, race, SES, social connectedness, health behaviours, education, BMI). After identifying that sex/gender was the most commonly recorded of these social categories, we focused further analyses on how sex/gender was or was not addressed in research design, analysis, and interpretation. To do this, we re-read and re-extracted the following data from all papers: inclusion/exclusion criteria, whether the study topic was sex-specific, number and proportion of women and men in the study, and how sex/gender was addressed and reported in results and discussion. A narrative summary of methodological strengths and shortcomings overall was also documented for each study.

## Results

The 57 trials reviewed are summarised in Table 1 (20–76). In total, six papers reported on sex-specific conditions (36, 47, 48, 58, 68, 74). Table 2 identifies social characteristics documented across the reviewed papers. Most frequently recorded was sex/gender (52/57, 91%), followed by race or ethnicity (30/57, 53%), or some aspect of SES (9/57, 16%). Although BMI, a possible proxy for SES, was sometimes recorded its use was solely as a physical indicator of risk. Social connectedness and past or present adversity, both known determinants of health, were not documented in the reviewed trials. Thirty-five studies (61%) had age-related exclusions, few of which were necessary given the condition being assessed. One trial excluded women without explanation. In this study of diabetes, subjects recruited through primary care practices were all men (39). Conversely, in what was designed as a sex-specific study of the impact of peer support for mothers on maternal and child morbidity and mortality in Malawi, researchers allowed men to participate in women's support groups (47). Pregnant women, or those who might become pregnant, were excluded in 13 of the 54 studies (24%) of either women alone or both men and women.

**Table 1:** Summary of reviewed RCTS

Author, Funding (public, industry, both), (reference number)	Aim/Question	Outcome	Population characteristics number, sex, race/ethnicity, age, measure of SES	Additional notes on method
Aberele (industry) (20)	Comparison of low dose CT and x-ray screening for detection and survival of lung cancer	Earlier detection with CT but also more false positives Decrease in the number of advanced-stage cancers detected	N>53,000 Women=39% Documented race, marital status, education, BMI, workplace risk	Compared participants to overall population to show external validity
Anderson (public) (21)	Comparison of Dalteparin versus ASA for extended DVT prophylaxis after hip arthroplasty	ASA was equally beneficial and much less costly	*N*=785 Women=43% Documented BMI, smoking	Reported as non-inferior
Boyd (primarily industry) (22)	Comparison of two drug regimens for HIV after failure of first-line treatment	Found no benefit to new treatment	*N*= 558 Women=47% Age >16 Documented ethnicity, sexual orientation	Reported as non-inferior
Burmester (industry) (23)	Comparison of methotrexate+or – tofacitinib for second-line treatment of RA	Combination treatment was effective	*N*=399 Women=84% Documented race	Phase 3 drug trial
Byrd (industry) (24)	Comparison of two doses of a drug to treat CLL	Outcome described drug as effective but without a control group	*N*=85 Women=24%	Protocol changed during study Preponderance of men 34% attrition from study – unclear if they dropped out or were removed or whether they were included in analyses Endpoints unclear
Chakravarthy (public) (25)	A comparison of two drugs to treat macular degeneration	Outcomes were similar for each group but one drug was less costly	*N*=628 Women=60% Age >50 Documented quality of life, depression	Unclear why study included depression as an independent variable
Cooper (public/ industry) (26)	Comparison of medication alone or with stenting for renal artery stenosis and hypertension or chronic renal disease	No difference in outcomes	*N*=931 Women=50% Age 18+ Documented race, BMI, smoking	Pre-specified secondary analyses of interactions with sex, race, and medical variables
Cotton (public/industry provided drugs) (27)	Comparison of early versus deferred anti-retrovirals for infants with HIV	Early treatment improved long-term outcomes	*N*=377 Sex not specified Age >12 weeks	Could have compared boys and girls to assess differences in immune response
Dennis (public/industry) (28)	Does intermittent pressure reduce DVT risk days to weeks post-stroke better than no treatment?	Absolute risk reduction 3.6% with more skin problems in treatment group	*N*=2,876 Women ~50% Documented ‘overweight’ and ‘lives alone’	Not blinded
Devanand (public) (29)	Does stopping risperidone precipitate psychosis among Alzheimer's patients?	Increased psychosis when drug stopped	*N*=110 Women=60% Age 50–90 Documented race, place of residence	
Doody (industry) (30)	Comparison of drug versus no treatment for Alzheimer's disease	Treatment group had worse outcomes and more side effects	*N*=1,537 Women=54% Age <55	Phase 3 drug trial Many (~1/3) subjects removed from study despite meeting inclusion criteria
Dooley (industry) (31)	Comparison of mycophenolate versus azathioprine for lupus-induced renal failure	Mycophenolate significantly better for all outcomes	*N*=227 Women=86% Age 12–75 Documented weight, urban/rural residence	Phase 3 drug trial 45% withdrawal secondary to side effects (sex undisclosed)
Douillard (industry) (32)	Comparison of new and conventional treatments for colorectal cancer based on genetic markers	Outcomes were associated with presence of gene mutations	*N*=620 Women=30% Age 18+ Documented race, ethnicity	
Elkenboom (industry) (33)	Comparison of dabigatran and warfarin in patients with mechanical heart valves	Excess thrombolysis and bleeding in dabigatran group such that study was terminated	*N*=252 Women=35% Age 18–75	Reported a harmful outcome from the study drug and terminated the study early
Estruch (primarily public) (34)	Can a Mediterranean diet prevent cardiovascular disease among those at high risk?	Diet was effective	*N*=7,447 Women=57% Age 55–80 (men) and 60–80 (women) Documented BMI, race, smoking	Despite randomisation there were baseline differences in groups
Fayad (industry) (35)	Comparison of dalcetrapib versus placebo on atherosclerosis	No significant benefit	*N*=130 Women=18% Age 18–75 Documented race (white=93%)	Preponderance of men suggesting inclusion bias
Fleshner (industry) (36)	Comparison of dutasteride versus placebo in low-risk prostate cancer	unclear	*N*=289 Single sex condition Age 48–81 Documented race, BMI, anxiety regarding cancer	Complex statistical analyses to look for some benefit for the test medication
Forster (public) (37)	Comparison of structured education program to none for informal caregivers of stroke patients	No differences in patient outcomes	*N*=928 patients Women=44% *N*=928 caregivers Women=68% Documented race, education, employment	Comments in paper re excess of women as caregivers
Gebre (public) (38)	Comparison of twice- or once-yearly treatment for trachoma	No significant difference in outcome	*N*=33,190 Women=48% Age >9 Documented distance to hospital	Documented that only predictor of greater infection was greater proportion of girls/women in cluster but did not discuss potential issue of gender equality
Griffin (public/industry) (39)	Comparison of the impact of less or more intensive treatment of diabetics on CVD after 5 years	No significant difference in outcome	*N*=3,057 Women=0% Age 40–69 Documented race (white=96%), unemployment	No explanation for how recruitment of diabetics via GP practices yielded no women Authors call theirs a representative, population-based sample No mention of lack of women in discussing external validity
Hoffman (public) (40)	Does emergency room use of coronary CT angiography decrease time in hospital for chest pain?	Time in hospital decreased but later testing and radiation increased	*N*=1,000 Women=47% Age 40–74 Documented race, BMI	
Horton (industry) (41)	Cross-over study of thalidomide for cough in severe idiopathic pulmonary fibrosis	Treatment improved cough and respiratory quality of life	*N*=23 Women=22% Age >50 Documented race	
Kang (public) (42)	Comparison of early surgery versus standard treatment for infective endocarditis	Intervention decreased embolic events and death	*N*=76 Women=33% Age 18–80	
Karim (public) (43)	Comparison of timing of antiretroviral and TB treatments to prevent AIDS progression	No significant differences arising from timing	*N*=429 Women=51% Age >16 Documented education, autonomy	Noted some sex-specific side effects, e.g. cervical dysplasia, menorrhagia
Katz (public) (44)	A comparison of partial meniscectomy versus physiotherapy for symptomatic meniscal tears	No real difference in outcome by intention to treat but 30% of physio group underwent surgery	*N*=330 Women=57% Age >44 Documented race, BMI, mood	Not blinded 83% of those assessed were not included, reasons not clear
Kolle (public/industry) (45)	Comparison of expanded adipose tissue derived stem cells and ordinary fat grafts	Better graft survival with stem cells at 121 days	*N*=10 Women=90% Age 18–45	
Lebwohl (industry) (46)	Comparison of a new gel (Ingenol mebutate) with other treatments of actinic keratosis	New treatment was no better than existing ones	*N*=1,005 Women=25% Age >18 Documented race (white=100%)	Could have commented that actinic keratosis more common and reported elsewhere to be more treatable in men
Lewycka (public and NGOs) (47)	Does peer health education of mothers improve breastfeeding rates and lower mortality in rural Malawi?	Unclear – overall – no significant impact	Cluster randomisation: 48 clusters, 26,262 births (?) Age of women: 10–49 Recorded tribe, education, occupation, religion, marital status, SES, parity	Designed as a single sex study but men included in groups 20% lost to f/u Selected data used to find some benefit to intervention
Liem (public) (48)	Can pessary insertion at 16–20 weeks’ gestation decrease preterm birth in multiple pregnancy	Pessary did not decrease risks and did increase vaginal discharge	*N*=813 Single sex condition Documented race, BMI, education	Not blinded
Lo-Coco (primarily public, drugs donated by industry) (49)	Comparison of two drug regimens to treat promyelocytic leukaemia	New drug treatment was no better than old treatment	*N*=156 Women=51%	Reported as non-inferior
Mallick (public/industry) (50)	Comparison of low- and high-dose radioiodine for treating thyroid cancer	No difference in effect and lower dose had fewer side effects	*N*=438 Women=74% Age 16–80	
Marchioli (industry) (51)	Comparison of phlebotomy, hydroxurea or both for treatment of lower or higher hematocrits in polycythemia	Those with lower hematocrit had significantly better outcomes, i.e. fewer deaths from cardiovascular or thrombotic events	*N*=365 Women=38%	No comment on whether smaller hematocrit elevations have greater impact on health of women
Mateos (industry) (52)	Comparison of drug to no drug for smouldering multiple myeloma	Drug more effective than no treatment	*N*=119 (10% withdrawn from study by researchers) Women=55%	Phase 3 drug trial Only 42% completed study
Milstone (public/ industry) (53)	A comparison of chlorhexidine and regular baths to decrease bacteraemia in critically ill children	No statistically significant difference found	*N*=4,947 sex not specified age <2 months Documented race	Not blinded 371 in treatment group excluded for lack of consent versus none excluded in control group Enrolment biases identified
Montalescot (industry) (54)	Does prasugrel prior to rather than at the time of angioplasty post-non-STEMI decrease mortality?	Outcome: no benefit of pre-treatment and greater harm from life-threatening bleeding	*N*=4,033 Women=28% Documented race: (Caucasian=97%), BMI, health behaviours	Phase 3 drug trial Supplemental data says women more likely than men to bleed (*p*=0.09) – not reported in paper, itself No explanation for low enrolment of women
Motzer (industry) (55)	A comparison of two chemotherapies for metastatic renal cancer	Trial drug was no better than control	*N*=1,110 Women=27% Geographic and ethnic subgroups used for analyses	Trial drug reported as ‘non-inferior’ No documented exclusion criteria yet 300 excluded Protocol changed mid-study Often reported numbers without statistical significance
Nguyen-Khac (public) (56)	Comparison of Prednisolone with and without *N*-acetylcysteine for severe alcoholic hepatitis	No benefit to 2 drug regime	*N*=174 Women=40% Age >18	Not blinded
O'Dell (public) (57)	A cross-over comparison of 2 second-line treatments for RA	New treatment was no better than old	*N*=353 Women=46% Age >17 Documented race, BMI, smoking	Study treatment reported as non-inferior Although women responded better to new treatment than expected and similar to men's response, didn't comment on this
Parker (industry) (58)	Assessed efficacy and safety in treating metastatic prostate cancer with radiation vs. placebo	Life expectancy with radiation versus placebo: 14.9 versus 11.3 months.	*N*=921, single sex condition Documented race, Many medical exclusions	-reported relative risk reduction (30%) rather than absolute
Pickard (public) (59)	Comparison of antimicrobial and regular urethral catheters for short-term use	No significant difference in infection rate	*N*=6,394 Women=62% Age >15	Not blinded No consideration of whether anatomical differences might lead to differences in infection risk for men and women that could have been seen by disaggregating data
Pirmohamed (public) (60)	Comparison of genotype guided and standard dosing of warfarin for atrial fibrillation or DVT	Genotype-guided dosing reduced number of days with high INR and time to steady state dosage	*N*=455 Women=39% Documented race, BMI, smoking	Treating physician could exclude subjects for undefined reasons
Roberts (industry) (61)	Does point of care genetic testing identify those for whom clopidogrel presents a high risk?	Effective identification of carriers of gene	*N*=187 Women=22% Documented race, weight, smoking	
Roe (industry) (62)	Comparison of prasugrel versus clopidogrel for acute coronary syndromes without re-vascularisation	No significant differences found	*N*=9,326 Women=39% Documented race, weight, smoking	Large enrolment would have allowed for assessing sex differences in side effects like bleeding, but not done
Shaukat (public) (63)	Comparison of long-term outcomes among those undergoing faecal occult blood screening for colon cancer	After 30 years those screened had significantly lower mortality from colon cancer but not lower overall mortality	*N*=46,551 Women=52% Age 50–80	Assessed sex-specific relative risks of dying from colon cancer
Sihvonen (public) (64)	Comparison of partial meniscectomy and sham operation for degenerative meniscal tear with no osteoarthritis.	No differences in outcomes found	*N*=146 Women=39% Age 35–65 Documented BMI	
Silbergleit (public) (65)	Comparison of IM midazolam versus IV lorazepam after 5 minutes of seizures	Midazolam of significant benefit	*N*=893 Women=43% Documented race (African Am.=51%)	Phase 3 drug trial
Soofi (NGO/industry) (66)	Can 1 year of zinc+or – micronutrients improve growth and limit infection and diarrhoea in infants	Supplementing with zinc decreased Fe deficiency anaemia but increased side effects	*N*=2,746 Sex unspecified Documented water source, sewage system, household income	Could have considered whether giving supplements might change food offered to infants and particularly to girls
Thiele (public/industry) (67)	Comparison of intra-coronary versus IV abciximab post-STEMI	Of multiple endpoints only heart failure might be improved with intra-coronary treatment	*N*=2,065 Women=25% Age <75 Documented race (all ‘white’)	Not blinded Although women had significantly better outcomes than men this was attributed to chance Enrolment bias noted
Tylleskar (public) (68)	Does peer counselling mothers of infants decrease diarrheal disease by increasing breast feeding in Africa?	No significant decrease in diarrhoea despite increase in breast feeding	*N*=2,579 Sex-specific study Documented education, marital status, SES, electricity in home, water source, toilet, parity, previous child death, antenatal care, place of birth, BMI	Data on main outcome downplayed and instead authors conclude breast feeding must be beneficial and might shorten course of diarrhoea despite no evidence for this
Von Hoff (industry) (69)	Comparison of new and standard drug combinations for treating pancreatic cancer	New combination prolonged survival by about 2 months	*N*=861 Women=42% Age 18+ Documented race and region	Phase 3 drug trial
Wang 98b (public) (70)	Comparison of ASA+or – clopidogrel for stroke prevention after TIA	Additional drug decreased stroke without increasing haemorrhage	*N*=5,170 Women=34% Age 40+ years BMI, smoking	Interactions assessed in subgroups and documented
Walmsley (industry) (71)	Comparison of new and older combinations of treatments for HIV	New combination reported as more efficient and safer	*N*=833 Women=16% Age >18 Documented race	Preponderance of men was commented on by authors as arising from exclusion of pregnant or potentially pregnant women
White (public/industry) (72)	Comparison of alogliptin versus placebo in diabetics hospitalised for unstable angina or MI	Drug was found to be no better than placebo	*N*=5,830 Women=32% Documented race, BMI, smoking, region	Reported outcome as non-inferior Stratified results by region Reported sex stratification in appendix
Wiviott (industry) (73)	Comparison of prasugrel and clopidogrel post-non-STEMI and without re-vascularisation	Unclear – reported that test drug was beneficial	*N*=7,243 Women=36% Age <75	Phase 3 drug trial No explanation for disproportion of men Women were less likely to be investigated with angiography
Young (public) (74)	Can social media networks increase HIV testing among African American and Latino men who have sex with men?	Intervention increased HIV testing	N122 Single sex study Age 18+ Documented race, education, birthplace, income social connectedness, urban/rural, sex behaviours	
Zeuzem (industry) (75)	Comparison of faldaprevir plus deleobuvir at different doses, with or without ribaviron to induce a sustained virologic response in chronic Hep C	Including Ribavirin improved outcome	*N*=362 Women=48% Documented age, race, BMI	Phase 2 drug study Not blinded Initial trial halted by FDA Changed protocol during study and redefined endpoint
Zhu (public/industry) (76)	Comparison of vaccine versus placebo to prevent enterovirus infection	The vaccine was effective and produced no more side effects than placebo	*N*=10,245 Girls=44% Age 6–34 months Documented BMI	Phase 3 trial

**Table 2:** Inclusion/exclusion of individual characteristics in reviewed RCTS (*N*=57)

	Age	Sex/gender	BMI	Health behaviours	Race, ethnicity	SES, education	Other individual characteristics
*In demographics*	23, 26, 27, 29, 33, 37, 40, 41, 42, 45, 46, 50, 51, 59, 60–64, 69, 71, 72, 74	20–26, 28, 29–31, 33–35, 36 (sex-specific condition), 37–46, 48, 49–52, 54–57, 58 (sex-specific disease), 59–65, 67, 68 (sex specific), 69–76	20, 21, 26, 28 (overweight noted), 31 (weight), 34, 36, 40, 44, 48, 54,57, 61 (weight), 62 (weight), 64, 68, 70, 72, 73, 75, 76	21 (smoker), 26 (smoker), 34 (smoker), 47, 54, 57 (smoker) 60 (smoker), 61 (smoker), 62 (smoker), 70, 72 (smoker), 74	20, 22, 23, 29, 31, 34,35 (93% white), 36, 37, 39 (96% white), 40, 41, 44, 46 (all white), 47, 48, 53, 54, 57, 58, 60–62, 65, 67 (all white), 69, 71, 72, 74, 75	20, 37, 39, 43, 47, 48, 66, 68, 74	22 (sexual orientation), 25 (quality of life, depression), 28 (lives alone), 29 (residence), 31 (location), 36 (anxiety re cancer), 37 (employment), 38 (location), 43 (Karnofsky score), 44 (mental health), 47 (social connectedness, marital status), 55 (site), 68 (marital status, community traits), 69 (region), 72 (world region), 74 (birthplace, social connectedness, urban/rural)
*Among explicit exclusion criteria*	20, 22, 23, 25, 27 (infant study), 28, 29, 31,33–36, 38–47, 49, 50, 52, 53, 56, 57, 63, 64, 66, 70, 73, 76	26 (pregnancy), 32 (pregnant), 36 (sex-specific condition), 39 (no women although not an explicit exclusion), 41 (pregnancy potential), 45 (pregnant), 47 (failed to exclude men), 48 (sex-specific condition), 51 (pregnancy or no contraception), 57 (women without contraception), 60 (pregnancy), 61 (pregnancy), 65 (pregnancy), 67 (pregnancy), 68 (breastfeeding study), 70 (pregnancy), 71 (if no proof not pregnant), 72 (planning pregnancy), 74 (women)	34 (>40), 40, 45	20 (non-smokers), 34 (substance abuse), 45 (smoker), 51, 57 (substance abuse), 72 (drugs, alcohol abuse)		34 (literacy), 65 (prisoners)	21 (distance to study centre), 30 (none stated but >1/3 withdrawn by industry researchers during trial without explanation), 31 (no exclusions but more than ½ withdrew due to side effects), 32 (no contraception for either men or women), 34 (possible difficulty following a diet), 44 (English illiteracy), 47 (sterilisation), 52 (none named but >10% of treatment group withdrawn by researchers without explanation), 53 (5–10% withdrawn overall but all from treatment group for lack of consent), 55 (none named but 300 excluded), 65 (weight of children, also more than 2/3 excluded for unnamed reasons), 60 (treating MD determined eligibility),
							67 (medical conditions), 72 (investigators’ opinion re debility), 75 (1/4 of those screened excluded, without explanation)

Table 3 documents whether and how sex/gender was addressed in the studies reviewed. We considered papers where women and men were included in proportions ranging from 40 to 60% as having equal representation. Of the 51 non-sex-specific trials, 20 (39%) enrolled women and men in roughly equal proportions, 22 (43%) included more than 60% men, in 5 (10%) more than 60% of participants were women, and sex/gender proportions were not documented in three studies conducted among adults and two among children. Not noted in Table 3 is that the majority of papers in the sample used the concepts ‘sex’ and ‘gender’ as undefined synonyms when describing inclusions, exclusions, results, and in discussions.

**Table 3:** Addressing sex/gender in reviewed RCTS (*N*=57)

	Yes (reference number)	No (reference number)
Sex-specific condition^a^	36, 47,48, 58,68 74 (although HIV not sex-specific target population was gay men)	20–35, 37–46, 49–57, 59–67, 69–3, 75,76
Information on sex/gender of participants documented	20–26, 28–30, 33–35, 37–46, 49–52, 54–57, 59–65, 67, 69–73, 75, 76	27, 31 (proportions of overall cohort reported but not of those who completed trial), 32 (incomplete numbers and only in appendix), 53 (children), 66 (children)
Women and men in non-sex-specific studies (*n*=51)		
Overall	20–35, 36–38, 40–46, 49–57, 59–67, 69–73, 75, 76	39
Proportions of men and women stated	20–6, 28–30, 32 (info in appendix only), 33–35, 37–46, 49–52, 54–57, 59–65, 67, 69–73, 75, 76	27, 31 (noted proportions of whole group but not of group that was analysed and completed study), 53, 66
Disproportion of men to women, i.e.>or=60:40	20, 24, 32, 33, 35, 39, 41, 42, 46, 51, 54–56, 60–62, 64, 67, 70–73	22, 23, 25, 26, 28–30, 34, 37, 38, 40, 43–45, 49, 50, 52, 57, 59, 63, 65, 68, 69, 75, 76
Disproportion of women to men, i.e.>or=60:40	23, 37 (assuming caregivers were primary participants, rather than patients), 45, 50, 59	20–22, 24–26, 28–30, 32–35, 38–44, 46, 49, 51, 52, 54–57, 60–65, 67, 69–73, 75, 76
Proportion of men to women approximately equal, i.e. each at least 40% of sample	21, 22, 25, 26, 28–30, 34, 38, 40, 43, 44, 49, 52, 57, 63, 65, 69, 75, 76	20, 23, 24, 32, 33, 35, 37, 39, 41, 42, 45, 46, 50, 51, 54–56, 59–62, 64, 67, 70–73
Sex/gender included in analysis and/or discussion (*n*=57)^b^		
Overall	20, 26, 34, 38, 62, 63, 69–72, 75	21–25, 27–33, 35–37, 39–61, 64–68, 73, 74, 76
Via data disaggregation (separate analyses for women and men)	20, 34, 63, 67 (mentioned subgroup analyses), 69–71, 72 (compared hazard ratios in appendix)	21–33, 35–62, 64–66, 73,–76
As an independent variable	26, 38 (proportion of girls in each cluster) 62 (in appendix), 69, 71, 75	20–25, 27–37, 39–61, 63–68, 70, 72–74, 76
Via interaction with other variables	26, 63, 70	20–25, 27–33, 34 (could have examined gender via known interaction of sex*BMI), 35–62, 64–69, 71–76
Sex/gender included in interpretation/ discussion	20, 48 (commented re benefit of a low cost intervention), 51, 54 (comment in supplement that women had more adverse outcomes), 57 (one statement), 60 (disproportion of men mentioned), 63, 67 (dismissed significant gender differences as likely due to chance), 71 (disproportion of men mentioned, as was same outcomes both sexes)	21–25, 26 (despite excellent methodology), 27–46, 47 (confusing analysis but didn't appear to discuss social traits), 49, 50, 52, 53, 55, 56, 58, 59, 61, 62, 64, 65, 66 (no consideration of whether girl children might be systemically disadvantaged, i.e. whether giving supplements might precipitate parental redirecting of food to other children), 68 (one statement that SES was not a confounder), 69, 70, 72–76

a Conditions affecting either men or women but not both.

b Includes studies of sex-specific conditions.

Explicit and implicit methodological aberrations were not uncommon (see Table 1) particularly with respect to selection of participants. In five papers, for example, there were unclear reasons for exclusions or high numbers of unexplained dropouts among those already enrolled (24, 30, 44, 52, 55). In a different study, 371 (~25%) of those randomised to the treatment group yet none in the control group were unavailable to consent and were therefore excluded (53). In another study almost 80% of recruits were removed (44). We noted that arbitrary and ill-defined options to exclude participants at intake or after were more common in trials with some or all funding from industry (90%) (24, 30, 39, 52–55, 60, 73) than solely from public sources (44). For five of the eight trials that were not blinded, there was no reason related to the intervention itself that would preclude this standard methodology (48, 53, 56, 59, 75).

Next we examined whether and how sex/gender differences were analysed and included in results. Of the 49 of 51 studies on non-sex-specific conditions that included both women and men, only 10 (20%) used these categories to differentiate findings either via disaggregating data (*n*=8) (20, 34, 63, 67, 69–72) and/or by examining interactions between sex/gender and other variables of interest (*n*=3) (26, 63, 70).

Aspects of sex/gender that were described in outcomes were sometimes discussed (20, 51, 57, 63, 67, 71). Also, in one of the six studies of sex-specific conditions there was discussion of whether gender aspects like social roles, opportunities, constraints, and expectations inherent in being a woman might interact with findings (48). Conversely, identified differences between women and men were, on occasion, not reported in results but alluded to in subsequent discussions (48, 60) or in appendices (54). Overall, the interactions between sex/gender and other social determinants of health (e.g. SES, education, race) that would enrich understanding were neither included nor discussed as missing explanatory indicators, possible sources of error, or as predictors of the outcome.

## Discussion

In this systematic sampling review of recent RCTs, we have assessed whether social traits exemplified by sex/gender were included in or potentially biased findings. Studies continue to show that women are underrepresented in enrolment (77, 78) in National Institutes of Health (NIH) funded clinical research despite funding guidelines (79–81) in research on specific diseases (82–84), in analyses (85), and when mortality is an endpoint (86). Our question went beyond inclusion of women to examine whether and how the impact of diversity in baseline social characteristics within study arms was addressed. We selected sex/gender for in-depth examination recognising that inclusion is a prerequisite for but does not alone address the social character of being men and women. It is because dissimilar characteristics of subgroups such as men and women can and do modify outcomes and should be addressed that major public funders are more and more insistent on inclusion (10, 19, 87). The European Commission website on Research & Innovations includes a Gendered Innovations section to support researchers by presenting examples of how to analyse sex and gender aspects in parallel (88). Such resources reaffirm that inclusion alone does not identify impact. Evidence of the differential effect of sex/gender on, for example, cardiovascular disease diagnosis, treatment offered, and prognosis (19, 89), or on pharmaco-dynamics in general is robust enough to recommend inclusion of sufficient numbers of participants to enable subgroup analyses for women and men (13).

Our focus was whether and how sex/gender was addressed in clinical trials rather than whether this social characteristic modifies outcomes for specific interventions or diseases. Whether social circumstances are acknowledged as integral to outcomes and elevated to the level of variables for analysis rather than controlled into oblivion is a matter of methodology across RCTs regardless of their specific topic. We therefore did not limit the sample to studies of particular systems and included all RCTs published in the journals and time frame selected. This yielded a mix of individual and cluster randomised trials; public and private funders; and pharmaceutical, technologic, and educational interventions. To the best of our knowledge, no study prior to ours has systematically assessed sex/gender in formulation of the research question, design, analysis, and interpretation in randomly selected studies from high impact medical journals. The composite picture arising is one of loss of information at each step in the path from design, through recruitment, data analysis and interpretation, a loss that can embed error in evidence.

### Research question and design

In general, men and women were included, although over-representation, usually of men relative to women, limited external validity for many of the trials reviewed. The preponderance of men in several studies of diseases with no male prevalence, studies that claimed random recruitment of participants, raises questions about interference in the selection process (54, 73) as did subjective and undefined exclusions (24, 30, 39, 44, 52–55, 60, 72, 73). In one study, the removal of almost 80% of recruits can only be assumed to introduce selection bias (44). As mentioned earlier, arbitrary and ill-defined options to exclude participants at intake or during the study were more common in trials with some or all funding from industry (90%) (24, 30, 39, 52–55, 60, 73) than solely from public sources (44). A European study of interventions to decrease cardiovascular mortality among diabetics did not mention why all participants were men. Being a woman was not among exclusion criteria, recruitment occurred in general practices where women are well represented, neither the interventions the disease studied nor the outcome were specific to men, and there was no indication in the title or abstract of the exclusion of women (39). This was the sole study to demonstrate a total blindness to sex/gender that was criticised widely and became a reason for denial of public funding more than 20 years ago in the United States (90). In contrast, a study of whether social networking can increase HIV testing among African- and Latino-Americans intentionally limited research on a non-sex-specific illness or intervention to men who have sex with men (74). By being explicit about reasons for selecting an all-male population, the authors minimised bias in their research question. The study by Lewycka et al. (47) on whether peer health education of women could increase breastfeeding rates and decrease morbidity or mortality in Malawi illustrates sex/gender bias via over-inclusion. Allowing men to attend peer groups for women in a setting where women generally lack autonomy created the potential for silencing or coercion of female by male participants. Put another way, having men participate in what should be a study of women's health education introduced potential bias arising from gender inequality. This was not considered in the paper.

### Analysis and interpretation

Data related to sex/gender, although available, were often neither utilised nor discussed. Authors might argue that such data served as evidence that randomisation controlled the impact of baseline characteristics like sex/gender via equal distribution and that funding limitations precluded powering studies to examine different outcomes for women and men. However, one could suggest that such an argument is evidence of, and will perpetuate blindness to the impact of sex/gender on health outcomes. There were no examples of a priori consideration of sex/gender by randomisation within the groupings ‘men’ and ‘women’ rather than in the sample as a whole. In the 8 studies where results were disaggregated for men and women these findings were generally not discussed and none considered reasons for sex/gender differences (20, 34, 63, 67, 69–72). This silence, although methodologically appropriate (because subgroup analysis was not planned a priori) occurred in studies where enrolment was adequate to identify differences and so, a research opportunity was lost. Three examples illustrate this. A study of new drug regimens for rheumatoid arthritis found women's responses equalled those for men (57). The authors noted that in prior research men had responded better than women to treatment, but made no comment about their novel findings and instead stated that there was no sex differential in response. In a study of interventions immediately following a STEMI (a kind of myocardial infarction) statistically significant differences in response by women and men were dismissed as a chance finding (67). Finally, although the only predictor of prevalence of trachoma infection was the proportion of women in each randomised cluster studied (*p*=0.002), this was ignored while statistically insignificant variability across treatments (*p*>0.99) was summarised as possibly of importance (38).

At times, authors’ statements did not match findings, putting a positive spin on interventions of no benefit or possible harm. New treatments that were no better than controls were termed ‘non-inferior’ (see comments in Table 1). Although overstating benefit was most common in tests of new drugs, two publicly funded studies highlight how unshakeable beliefs shaped reporting (47, 68). Both studies assumed that increasing breastfeeding rates would improve infant health in Africa. In each, the proportion of breastfed babies did increase; however, when there was no subsequent change in designated health outcomes authors hypothesised that there were likely other, non-measured advantages to breastfeeding. The evidence of no benefit from breastfeeding was, for whatever reasons, deemed unacceptable to report.

Social characteristics such as sex/gender are not always modifiers of the relationship between interventions and health outcomes. In treatment trials for endocarditis (42) or pancreatic cancer (69), sex/gender as a determinant or an effect modifier seemed unlikely. However, when sex/gender, race or SES matter will not be detected by randomising them into hiding. Without comment from authors it is impossible to determine what reasoning preceded limiting analyses to the group as a whole or if the impact of social circumstances was considered. Only by including subgroup analyses can researchers ascertain whether and when the measurement of social traits is relevant.

### Limitations

Drawing general conclusions about research methodology from analyses of 57 studies must be done with caution. However, by randomly selecting among the 712 RCTs identified, the sample studied should be representative of all papers identified in the initial search. In addition, interim findings after analysing 28 of 57 papers did not change substantially when all reports were included, making it seem that we had identified a pattern and could generalise from it.

One might argue it is difficult to analyse research from the varying medical areas included in this review in the same way. However, the point here was not to evaluate the relevance or best way to analyse sex/gender in one and each disease or condition. Instead, we searched for patterns and an overview of whether researchers seem aware of, and addressed, the fact that the groups men and women are not identical but instead often differ with respect to important biological characteristics and social and environmental living conditions.

## Conclusion

Few of the random samples of all RCTs published in five high-impact journals over 5 years assessed whether social circumstances altered outcomes. Fewer still attempted to interrogate findings with respect to sex/gender (or race or SES) and identify whether sex/gender acts as a modifier of the pathway from intervention to outcome. The theoretical robustness of the RCT is that it mimics animal experiments by controlling for, or randomly distributing and, therefore, removing unidentified and unmeasured human variability as sources of error. Baseline characteristics such as SES, race and sex/gender are, however, known determinants of health that can modify the relationship between exposures and outcomes of primary interest. Inherent in the methodology of clinical trials is the clean slate of equal background noise or impact of social characteristics like gender in all study arms but also the messiness of failing to hear the noise. It is only by listening by studying rather than randomising non-biological traits away, that research will identify whether and when social characteristics of participants affect outcomes.
